# Predictors of Surgical Site Infections among Patients Undergoing Open Urological Surgery at a Tertiary Hospital, Tanzania: A Cross Sectional Study

**DOI:** 10.24248/eahrj.v6i1.686

**Published:** 2022-07

**Authors:** Upendo O. Kibwana, Joel Manyahi, Victor Sensa, Sydney C Yongolo, Eligius Lyamuya

**Affiliations:** a Department of Microbiology and Immunology, Muhimbili University of Health and Allied Sciences; b Department of surgery, Muhimbili University of Health and Allied Sciences

## Abstract

**Background:**

Surgical Site Infection (SSI) is one of the major hospital acquired infections, highly associated with prolonged hospitalisation, morbidity and mortality. In open urological surgeries, little is known on magnitude and factors associated with development of SSI.

**Methods and Materials:**

The intervention was a cross-sectional prospective observational study performed between August 2015 and March 2016 at Muhimbili National hospital (MNH), Dar es Salaam, Tanzania. Patients who underwent open urological surgery at MNH during the study period and met inclusion criteria were consecutively enrolled, and followed up for 30 days. Patients' and operative characteristics were recorded using standard structured questionnaires. Wound/pus swabs were collected from patients with clinical evidence of SSI for bacteriological processing. Data analysis was performed using SPSS version 20.

**Results:**

Of 182 patients who underwent open urological surgery, 22% (40/182) developed SSI. Pre-operative urinary tract infection (aOR 9.73, 95%CI 3.93-24.09, *p<.001*) and contaminated wound class (aOR 24.997, 95%CI 2.58-242.42, p = .005) were independent predictors for development of SSI. Shaving within 30 hrs before surgical procedure was found to be protective for developing SSI (aOR 0.26, 95%CI 0.09-0.79, *p=.02*). *Escherichia coli* (20/40) was the most predominant pathogen in SSI followed by *Klebsiella pneumoniae* (7/40) and *S. aureus* (6/40). Gram-negative bacteria were highly resistant to ceftriaxone, gentamicin, amoxicillin-clavulanic acid and trimethoprimsulfamethoxazole.

**Conclusion:**

Surgical Site Infection was high in open urological interventions. Pre-operative urinary tract infection and contaminated wound class predicted SSI. Bacteria causing SSI were highly resistant to commonly used antibiotics.

## BACKGROUND

Surgical Site Infection (SSI) is among the major hospital acquired infections in patients undergoing surgical procedures. It is highly associated with prolonged hospital stay, increase in health care costs and mortality.^1,2^ Certain patient and operative (preoperative, operative and postoperative) characteristics have been documented to be contributing to the risk of SSI development.^3,4^

Despite improvement in infection prevention and control practices in surgical procedures, such as; improved operating rooms, trained medical personnel, sterilisation techniques and provision of surgical antimicrobial prophylaxis, SSI remains a common hospital acquired infection and this has significantly limited the realisation of the valuable effects of surgical interventions. The incidence of SSI is approximately four times higher in Low and middle-income countries than in high-income countries.^4^

Depending on geographical location, the rates of SSI in developing countries accounts between 10.9 and 26% among patients undergoing major surgeries.^5-7^ In addition, appreciable morbidity and mortality is attributed to SSI.^1,8^ In Tanzania, studies have reported surgical site infections rate of 24% and 19.4% in district and tertiary hospitals respectively.^6^

Determining the magnitude and identifying risk factors associated with the incidence of SSI in preoperative and operative stage is critical for prevention and control of SSI. There is limited data about predicting factors for SSI incidence among patients admitted in general surgical ward, and among pregnant mothers undergoing caesarean sections in Tanzania.^6,9^ Also, there is hardly any data about factors associated with SSI incidences among patients undergoing open urological surgery. In this study, we performed a cross-sectional observation study to determine the rate and predictive factors for SSI, aimed at establishing evidence-based preventive protocols against SSI at Muhimbili National Hospital and other settings with similar context.

## METHODS

### Study Design and Settings

This was a cross-sectional prospective observational hospital-based study conducted between August 2015 and March 2016 at the urology ward of Muhimbili National Hospital (MNH), located in Dar es Salaam, Tanzania. MNH is the main specialised tertiary and training hospital for Muhimbili University of heath and Allied Sciences. It has 1500-bed capacity and attends 1000 to 1200 outpatients weekly. It serves approximately 6 million people living in and around Dar es Salaam and Pwani regions. The urology unit has approximately 70 bed capacity and performs at least 5 urological procedures daily.

### Sample Size Estimation

The sample size calculation formula for finite population was applied to calculate the minimum required sample size


$$\text{n}=\frac{{\text{Nz}}^{\underline{2}}\text{pq}}{{\text{d}}^{2}(\text{N}-1)}+{\text{z}}^{2}\text{pq}$$

where N=size of population from observational pilot study for patients who underwent open urological surgeries at MNH; about 256 underwent open surgeries for a period of 8 months

n=sample size

d= margin of error at 95% Confidence Interval which was considered to be 3%

q=1-p

p = proportion of patients with surgical site infection in general surgery at MNH which is 15.6% (add ref).

After adjusting for the non-response rate at 10% the minimum required sample size was 195 patients. However, a total of 212 patients were recruited during the study period.

### Study Population

All patients who underwent open urological procedure at Muhimbili National Hospital during the study period and consented to participate in the study were included in the study and were followed up for a period of 30 days. Patients who were malnourished and those with chronic illnesses such as HIV were excluded from the study. Surgical wounds were examined 48 hours after surgery and thereafter on each day until the patient was discharged. At the 7^th^ day, during suture removal, the wound was examined again and whenever required, until day 30. During examination, the attending surgeon determined whether the patient had signs and symptoms of SSI. Clinical and microbiological evidence of SSI was defined as previously described by the Centre for Disease Control and Prevention.^3^ A total of 212 patients underwent open urological surgery during the study period, 182 patients were followed for a period of 30 days, 9 patients died before completion of follow up period and 21 patients were lost to follow up.

### Data Collection

A Standard structured questionnaire adopted from a previous study conducted in the same settings was used to collect information.^22^ Prior to using the questionnaire, it was pretested by using a pilot sample of 10 patients admitted in general surgery ward and had developed SSI. The questionnaire questions were asked in Tanzania's National Language (Swahili). Data collection was performed by registered nurses who were trained on the study protocol. Information recorded included sex, age, co-morbidities, history of cigarette smoking, number of days of hospitalisation before surgery, history of abdominal hair removal before surgery, class of surgical incision, pre-operative urinary tract infection, duration of surgery, and pre-catheterisation before operation.

### Specimen Collection and Laboratory Procedures

Patients with clinical evidence of SSI had two pus swabs or pus collected under aseptic procedure from the base of the wound and immediately transported to the laboratory in Amie's transport media (Oxoid, UK) for processing. Gram's stain was performed on the first swabs for assessing the quality of the specimens and stain morphology. When the first swab was of good quality, then the second pus swab or pus from wound was plated onto MacConkey agar (Oxoid, UK) and blood agar (Oxoid, UK) and incubated under aerobic condition at 37°C for 18 to 24 hours. Bacteria isolates were identified based on colonial morphology, Gram stain and a set of biochemical tests including Analytical Profile Index for Enterobacteriaceae (API 20 E).

Antibiotic susceptibility testing was performed using the Kirby Bauer's disc diffusion method, in line with the Clinical and Laboratory Standard Institute (CLSI) guidelines.

### Data Analysis

Data was entered and analysed using IBM SPSS software version 20, Armonk, NY IBM Corp. Data was summarised as; frequency, percentage and proportion. Binary logistic regression analysis was performed to identify the association between independent and dependent variables. Odds Ratios were used to test the strength of association between predictor variables. Significance was defined as a p-value of less than. 05.

### Ethical Considerations

Permission to carry out the study was obtained from Muhimbili University of Health and Allied Sciences, Senate Research and Publications Committee. Written informed consent was obtained from all study participants prior to enrolment. For participants below 18 years of age, informed consent was obtained from parents/guardians. Patient identification numbers were used instead of names to ensure confidentially.

## RESULTS

### Prevalence of Surgical Site Infection (SSI)

The rate of SSI in relation to various factors among patients who underwent open urological surgeries at MNH is summarised in Table 1. Of 182 patients who were followed up for 30 days, 40, 22% (95% CI 16.6 – 28.5) developed SSI. SSI was more common in males (32/144; 22.2%) and those aged between 54 and 71 years (13/47; 27.7%). Patients with diabetes had high rates of SSI (2/6; 33.3%) compared to patients with other co-morbidities. Interestingly, SSI was observed more among non-smokers (37/164; 22.6%) compared to cigarette smokers (3/18;16.7). The rate of SSI was higher in patients who were admitted for more than 3 days (18/50; 36%) compared to those admitted for lesser days. Patients who shaved more than 30 minutes before operation had high rate of SSI (9/23; 39.1%) compared to those who shaved within 30 minutes (12/38; 31.6%) and those who did not require shaving (19/21; 15.7%). Participants who had pre-operative urinary tract infections had increased prevalence of SSI compared to those without (26/44; 59.1% vs 14/138; 10.1%). SSI rate was more common (24/71; 33.8%) in catheterised patients compared to those who were not catheterised (16/111; 14.4%). Patients who's surgery duration was more than 90 minutes presented with higher rate of SSI compared to those who had less (33/127; 26% vs 7/55; 12.7%).

**TABLE 1:** The rate of SSI among Patients who Underwent Open Urological Surgeries between August 2015 and March 2016 at MNH

Variables	Total	Frequency	SSI (%)
**Age in years**			
<17	27	3	11.1
18-35	29	9	20.7
36-53	39	8	20.5
54-71	47	13	27.7
>71	40	7	28
**Sex**			
Male	144	32	22.2
Female	38	8	21.1
**Comorbidities**			
Diabetic mellitus	6	2	33.3
Hypertension	50	13	26.0
Diabetic mellitus+Hypertension	27	7	25.9
No comorbidities	99	18	18.2
**Cigarette smoking**			
Yes	18	3	16.7
No	164	37	22.6
**Days admitted**			
One day	69	14	20.3
2 days	47	7	14.9
3 days	16	1	6.2
>3 days	50	18	36
**Shaving**			
Within 30 minutes	38	12	31.6
>30 minutes	23	9	39.1
No need	121	19	15.7
**Catheterisation**			
inserted	71	24	33.8
Not inserted	111	16	14.4
**Pre-operative urinary tract infection**			
Yes	44	26	59.1
No	138	14	10.1
**Duration of surgery (Minutes)**			
<90	55	7	12.7
>90	127	33	26.0
**Type of wound**			
Clean	38	2	5.3
Clean contaminated	127	29	22.8
Contaminated	17	9	52.9

### Predictors of Surgical Site Infection

Shaving patients more than 30 minutes before operation was significantly associated with development of surgical site infections, cOR 3.451 (95%CI 1.308–9.105, *P=.012*). Having urinary catheterisation in situ before operation was significantly associated with surgical site infections on univariate analysis cOR 3.032 (95%CI 1.472 – 6.246, *p value .003*). The odds of SSIs were 12 times more in patients who had had pre-operative urinary tract infections before surgery (cOR 12.794, 95%CI 5.655 – 28.94, *p value <.001*). On univariate analysis, contaminated surgical procedures were found to be significantly associated with development of surgical site infection cOR 20.3, (95%CI 3.651 – 112.3, *p=.001*) (Table 2). Age, history of cigarette smoking, comorbidities, duration of admission before surgery and duration of surgery were found not be associated with the development SSI among patients who underwent urological surgery.

**TABLE 2:** Predictors of SSI among Patients who Underwent Open Urological Surgery between August 2015 and March 2016 at MNH

Variable	Number of patients	Rate of SSI n (%)	cOR	95%CI	P value	aOR	95%CI	P value
**Age (years)**								
<17	27	3 (11.1)	1					
18 – 35	29	9 (31.0)	2.087	0.466 – 9.346	0.336			
36 – 53	39	8 (20.5)	2.065	0.494 – 8.626	0.320			
54 – 71	47	13 (27.7)	3.056	0.785 – 11.915	0.107			
>71	40	7 (28.0)	2.667	0.659 – 10.786	0.169			
**Cigarette smoking**								
Yes	18	3 (16.7)	0.686	0.188 – 2.500	0.568			
No	164	37 (22.6)	1					
**Comorbidities**								
Diabetic mellitus	6	2 (33.3)	2.25	0.38 – 13.24	0.37			
Hypertension	50	13 (25.5)	1.54	0.68–3.46	0.25			
**DM + Hypertension**		27	7 (26.9)	1.66	0.61 – 4.53	0.33		
No comorbidities	99	18 (18.2)	1					
**Days admit pre-operation**								
1 day	69	14 (20.3)	1					
2 days	47	7 (14.9)	0.687	0.254–1.859	0.460			
3 days	16	1 (6.3)	0.262	0.032–2.155	0.213			
>3 days	50	18 (36.0)	2.210	0.970–5.034	0.059			
**Shaving**								
Within 30 minutes	38	12 (31.6)	2.478	1.068–5.747	0.035*	0.26	0.09-0.79	0.02*
>30 minutes	23	9 (39.1)	3.451	1.308-9.105	0.012*	0.420	0.12–1.45	0.170
No need		121	19 (15.7)	1				
**Catheterisation**								
Inserted	71	24 (33.8)	3.032	1.472–6.246	0.003*	1.670	0.664-4.198	0.275
Not inserted	111	16 (14.4)	1					
**Pre-operative urinary tract infection**								
Yes	44	26 (59.1)	12.794	5.655–28.94	<0.001*	9.73	3.93-24.09	<0.001*
No	138	14 (10.1)	1					
**Wound class**								
Clean	38	2 (5.1)	1					
Clean contaminated	127	29 (23.0)	5.327	1.209 – 23.46	0.027			
Contaminated	17	9 (53.0)	20.250	3.651–112.3	0.001*	25	2.58-242.42	0.005*
**Duration of surgery**								
0 to 90 minutes	55	7 (17.5)	1					
>90 minutes	127	48 (33.8)	2.407	0.992–5.842	0.052			

SSI; Surgical site infection, cOR; Crude odds ratio, CI; Confidence interval, aOR; adjusted odds ratio

On multivariate analysis, pre-operative urinary tract infection was found to be independently associated with the development of surgical site infections aOR 9.73 (95%CI 3.93-24.09, p <.001). Contaminated surgical procedures were also found to be an independent factor associated with surgical site infection aOR 24.997 (95%CI 2.58-242.42, *p .005*) (Table 2). Shaving more than 30 minutes and urinary catheterisation were found to be not independently associated with surgical site infections and shaving within 30 minutes before surgical procedure was found to be protective against developing SSI aOR 0.26 (95%CI 0.09-0.79, *P=.02*).

### Bacteria Aetiology of SSI/UTI and their Antimicrobial Susceptibility Pattern

A total of 40 bacteria were isolated from patients with SSI, majority of the isolates were Gram negative bacteria. *Escherichia coli* were the most predominant pathogens causing SSI in 50% (20/40) patients undergoing urological surgery. *Klebsiella pneumoniae* accounted for 17.5% (7/40), *S. aureus* 15% (6/40), *Pseudomonas aeruginosa* 10% (4/40) and *Proteus mirabilis* 7.5% (3/340).

*Escherichia coli* isolates from SSI were highly resistant to ceftriaxone (84.2%), gentamicin (86%), amoxicillin-clavulanic acid (84%) and trimethoprimsulfamethoxazole (82.3%). Seventy eight percent (78%) of *Klebsiella pneumoniae* and 62% of *P. mirabilis* were resistant to ceftriaxone. *S. aureus* displayed high rate of resistance to amoxicillin –clavulanic (68.4%), while low rates of resistance were observed in ceftriaxone (42%), gentamicin (36%) and ciprofloxacin (27%).

## DISCUSSION

The overall prevalence of SSI among patients undergoing open urological surgery at a tertiary hospital in Tanzania was 22%. Our finding was higher compared to a study from developed countries like Serbia 5.9%,^2^ and a recent study in Egypt (9%) ^10^ among patients undergoing urological surgery. The contributing factors for the observed high rates of SSI in this study can be attributed to insufficient infection control and prevention measures. During this study's duration, wards were congested and patients who underwent surgery were mixed with other patients categories. In addition, most of the surgical procedure lasted more than 90 minutes, which also could have contributed to the increased rate of SSI. Conversely, the setting of this study was completely different from that in Serbia and Egypt, Tanzania being a developing country, with limited resources could count for the observed high prevalence rates. The rate of SSI reported in the present study was less than that reported by studies that considered general surgery,^6^ but higher than reports from studies that considered obstetrics surgeries in Northern, Tanzania.^9^ These findings from the same study settings suggest that patients' characteristics (risk factors) and surgical procedures determine the occurrences of SSIs. Patients from urology surgery might not share the same risk factors with their counterparts from general surgery and obstetrics.

Our study finding compares with reports from previous studies^11-13^, which reported that having urinary tract infections before urological surgical procedures is a risk factor for development of SSI among patients undergoing open urological surgery. Besides, SSI still developed in this study's participants with urinary tract infections being treated with antibiotics before surgery. This study could not ascertain if the same bacteria isolated from urinary tract infection were causative of SSIs, further molecular studies need to be performed to establish the clonal relationship. However, previous studies elsewhere^11,14^, documented that not all causative bacteria of SSI were of the same species as those from pre-operative urinary tract infections. Ideally, in a setting with culture facilities and elective surgery, urine culture needs to be performed before surgery.

Contaminated surgical incision was found to be a risk factor for development of SSI. This finding is consistent with reports of several previous studies performed in urology and general surgery wards.^2,4,10,15-17^ Several studies have reported correlation between wound class and development of SSI, the risk increases with classification of incision, surgeries on clean and clean – contaminated incision carry low risk compared to surgeries performed on contaminated and dirty incisions, which can be accounted for by high loading dose of microbes.^7,18,19^

Interestingly, shaving less than 30 minutes before surgical procedure on univariate analysis was associated with development of SSI. However, after controlling other factors, on multivariate analysis, shaving less than 30 minutes before surgical procedure was found to be a protective factor against the development of SSI. This observation is similar to findings from studies carried out elsewhere in different geographical settings^7,20^, which documented that increased time lapse between shaving and surgical procedure significantly increases risk for SSI.

The most common source of pathogens causing SSIs is endogenous from patients' own body flora. Bacteria are mainly transmitted to surgical sites during surgical manipulation. In this study, Gram negative bacteria such as *E. coli* and *Klebsiella pneumoniae* were the most common causes of SSI. Since all surgeries did not involve the opening of the gastro-intestinal tract, it was hardly unexpected to find predominance of these bacteria, which are flora of the gut. Due to close proximity of the rectum and site of incision, possibly the organisms may have ascended from the rectum. This study's results are consistent with results from reported by studies conducted among patients who underwent open urological procedures.^14,21^

Numerous studies in Tanzania have reported increased rate of antibiotic resistance among bacteria causing SSI.^6,22,23^ In our study, Gram negative bacteria causing SSI were highly resistant to ceftriaxone, which is commonly used for surgical antimicrobial prophylaxis. This is Consistent with reports from other studies conducted elsewhere.^22,24,25^, Bacteria resistance to third generation cephalosporin is on increasing trend in Tanzania, an observation that calls for further studies to assess the effectiveness of use of third generation cephalosporin as prophylaxis to prevent SSI.

This study was limited by the use of aerobic culture techniques and loss to follow up which could have cause under reporting of the reported prevalence.

## CONCLUSION

The rate of SSI among patients undergoing urological surgery at MNH was high. History of pre-operative urinary tract infections and contaminated wounds were found to be predictors for SSI. Most of Gram-negative bacteria isolated from SSI were highly resistant to ceftriaxone and other commonly used antibiotics. We therefore, recommend culture and sensitivity testing to patients on urinary catheter prior to surgical procedures, and improvement of IPC measures in the wards. Furthermore, evidence-based treatment approach using laboratory culture and sensitivity results to treat SSI should be used and establishment of surveillance system for SSI, so as to give appropriate feedback to the surgeons for patient management.
